# Investigation of vascular risk factor control and secondary prevention medication compliance in acute ischemic stroke

**DOI:** 10.3389/fneur.2024.1365860

**Published:** 2024-06-21

**Authors:** Yun Chen, Yuan Zhang, Lianyan Jiang, Yanbin Lu, Xiaojie Ding, Wei Jin, Canxin Xiong, Daping Huang

**Affiliations:** ^1^Zhejiang Provincial People's Hospital Bijie Hospital, Bijie, China; ^2^School of Clinical Medicine, Chengdu University of Traditional Chinese Medicine, Chengdu, China

**Keywords:** ischemic stroke, vascular risk factors, secondary prevention, medication compliance, clinical practice

## Abstract

**Objectives:**

This study aimed to investigate the management of vascular risk factors, with a specific focus on understanding the various factors affecting risk factor control through an in-depth analysis of clinical data and a longitudinal follow-up of patients who have experienced ischemic strokes.

**Methods:**

A total of 1,572 participants were included in the analysis. We assessed thresholds for blood pressure (BP), low-density lipoprotein cholesterol (LDL-C), and glycated hemoglobin (HbA1c) levels to uncover the contextual conditions and factors affecting vascular risk factor control. Moreover, the study also scrutinized medication compliance at intervals of 3, 6, and 12 months post-onset. Logistic regression was used to adjust for confounding factors.

**Results:**

At 3, 6, and 12 months, BP,LDL, hemoglobin control targets were achieved in 50.7, 51.8, and 50.6%; 51.5, 59.4, and 50.6%; 48.1, 44.0, and 48.4%,respectively. Notably, age was associated with the achievement of BP control (odds ratio [OR], 0.96; 95% confidence intervals [CI], 0.94–0.98; *p* < 0.0001). Ethnic minorities (OR, 4.23; 95% CI, 1.19–15.09; *p* = 0.02) and individuals with coronary heart disease (OR, 0.5; 95% CI, 0.3–1.0; *p* = 0.05) experienced decreased BP control ratios. A previous history of stroke (OR, 1.7; 95% CI, 1.0–2.8; *p* = 0.03) and unrestricted alcohol consumption (OR, 3.3; 95% CI, 1.0–11.1; *p* = 0.05) was significantly associated with the achievement of lipid control. Furthermore, lifestyle modifications were significantly correlated with the achievement of BP control (OR, 0.19; 95% CI, 0.12–0.30; *p* < 0.01), blood glucose control (OR, 0.03; 95% CI, 0.01–0.08; *p* < 0.01), and blood lipid control (OR, 0.26; 95% CI, 0.16–0.42; p < 0.01). The absence of regular physical activity was associated with lower rates of glycemic (OR, 0.14; 95% CI, 0.06–0.36; *p* < 0.01) and lipid controls (OR, 0.55; 95% CI, 0.33–0.90; *p* = 0.01). Over time, overall medication compliance declined.

**Conclusion:**

Within the cohort of patients under medication, the compliance rate concerning vascular risk factors remains unsatisfactory. Attention should be paid to compliance with secondary prevention medications and enhance the control of vascular risk factors, as compliance emerges as the key to effective prevention.

## 1 Introduction

Worldwide, stroke stands as the leading cause of mortality and disability, with its incidence showing a consistent increase (1–3). According to the statistics of 2019, China witnessed 30,000 new cases of stroke, indicating an 86% escalation since 1990 (4). Among stroke types, ischemic stroke emerges as the predominant variant, constituting 83% of all cases of stroke (5–7). Notably, recurrences within a year of onset vary between 10 and 17% (8). A comprehensive evaluation of stroke’s burden highlighted that effective risk factor control can improve >90% of the associated burden (9). In recent years, the effective use of secondary prevention medications like antiplatelet aggregation medications, antihypertensive, hypoglycemic, lipid-lowering, and anticoagulant medications has led to a decrease in the rates of recurrent ischemic stroke (10). Both international (11) and Chinese national guidelines (12) provide explicit recommendations for secondary stroke prevention. The primary focus for preventing the recurrence of stroke lies in managing related risk factors, including hypertension, hyperglycemia, and lipid metabolism disorders, followed by lifestyle modifications and regular physical activity.

Nevertheless, numerous studies have shown that these guidelines and recommendations exhibit limited implementation within clinical practice. This is evident not only through the observed low compliance with secondary prevention medications but also by the low compliance rates among patients who are prescribed medications to manage established risk factors (13). The diagnostic yield of hypertension among patients who have suffered from ischemic stroke is estimated to be approximately 70% (14–16). However, the control rate of hypertension remains relatively low at a rate of 15.3% (16). Treat Stroke to Target is a study to determine the impact of low-density lipoprotein cholesterol (LDL-C) levels on the recurrence of stroke in patients with atherosclerotic ischemic stroke (17). Therefore, the achievement of controlled LDL levels emerges as a pivotal element in preventing the recurrence of stroke.

The results of a meta-analysis highlight that approximately 28% of patients with stroke had diabetes mellitus, with a higher proportion (approximately 33%) observed among patients diagnosed with ischemic stroke (18). Within China, about 27% of hospitalized patients with ischemic stroke had diabetes (19). Several pieces of evidence from both domestic and international sources have consistently demonstrated a significant association between diabetes and adverse outcomes in the context of ischemic stroke. These outcomes encompass the initial occurrence of the stroke, its subsequent recurrence, and even mortality (19–22). The achievement of glycemic targets emerges as an imperative factor in the prevention of recurrence of strokes. A key to preventing the recurrence of stroke is steadfast compliance with recommended secondary prevention medications and the effective management of associated risk factors. However, various factors may interfere with medication compliance and hinder well-established risk factor control in patients with stroke (23). However, the exploration of how these factors precisely influence risk factor control and subsequently render the achievement of recommended targets relatively limited.

Compliance with secondary prevention medications among patients with ischemic stroke has been reported in China (24, 25). However, there is a paucity of study data pertaining to related vascular risk factor control, especially in the context of longitudinal follow-up. With the advancing age of the population and the ongoing updates to clinical guidelines, a pressing imperative exists to elucidate the practical implementation of secondary stroke prevention in clinical practice, along with the resultant benefits obtained. This study aimed to investigate the vascular risk factor and related factors affecting risk factor control in Guizhou, China, through an in-depth analysis of clinical data and a longitudinal follow-up of patients with ischemic stroke.

## 2 Materials and methods

### 2.1 Study population

This observational cohort study included patients with acute ischemic stroke who were admitted to the Department of Neurology at the First People’s Hospital of Bijie, Guizhou Province, between January 2019 and December 2020. And the study was approved by the local ethics committee. Inclusion criteria encompassed the following specifications: (1) fulfillment of the diagnostic criteria for ischemic stroke in the Neurology textbook (7th Edition, 2013), refers to a sudden onset of focal neurological dysfunction caused by local necrosis of brain tissue as a result of impaired blood supply to the carotid or vertebral base arterial system, and validated through head computed tomography or magnetic resonance imaging examination confirmation. (2) Age above 18 years; (3) diagnosis established within 7 days; and (4) volunteered to participate in this study.

**Figure 1:**
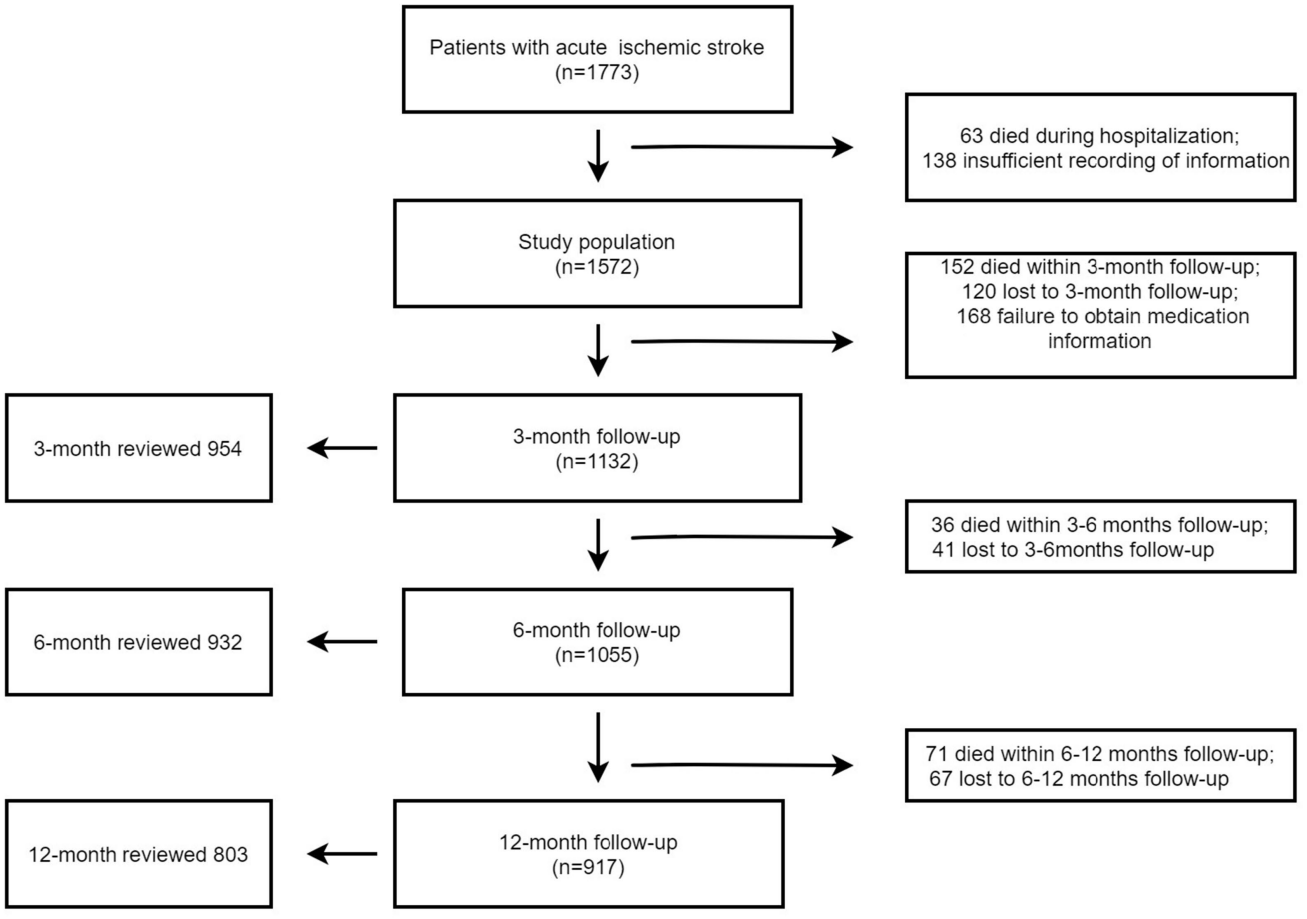
Flow chart of inclusion and exclusion of participants in current analysis.

A total of 1773 patients with acute ischemic stroke were initially enrolled. Those who experienced in-hospital mortality (*n* = 63) or were discharged without a prescribed medication regimen (*n* = 138) were excluded from the analysis, resulting in the inclusion of 1,572 patients (Figure 1). The demographic data such as age, sex, ethnicity (Han and minority nationalities); past medical history and clinical characteristics. Medication information upon discharge encompassed pertinent medications administered to patients without allergies or contraindications. This included antiplatelet, antihypertensive, hypoglycemic, lipid-regulating, and anticoagulant medications, with precise details recorded for each medication type, dose, and frequency.

Vascular risk factor control, medication usage, smoking and drinking habits, lifestyle modifications, and regular physical activity were recorded at 3, 6, and 12-month intervals. Participants who were unable to physically visit the hospital for re-examination were contacted by telephone for follow-up. To ensure the consistency of vascular factor control and minimize errors stemming from variations across different re-examination institutions, individuals in this category were not included in the analysis of vascular risk factor control. Instead, their records were limited to medication usage, smoking and drinking status, any adopted lifestyle modifications, and engagement in regular physical activities. In cases where participants faced difficulties responding due to conditions such as aphasia or deafness during the follow-up, pertinent information was provided by their family members or caregiving personnel. Patients who proved unreachable despite multiple attempts at communication were classified as lost to follow-up. The Ethics Committee of the Zhejiang Provincial People’s Hospital Bijie Hospital, Bijie, China, granted approval for this study (approval number: 3–1, year: 2023).

### 2.2 Outcome assessments

The primary outcome aligns with the treatment target recommended in the Chinese guidelines for secondary acute ischemic stroke prevention (12). This involves evaluating compliance rates in achieving the treatment targets for blood pressure (BP), LDL-C, and glycated hemoglobin A1c (HbA1c) control. Additionally, the study investigates factors that influence risk factor control, alongside other outcomes such as compliance with the prescribed secondary preventive medications upon discharge.

### 2.3 Risk factor control objectives

Baseline BP values were measured during hospitalization and in patients who returned for re-examination during follow-up. For these individuals, a neurologist employed a consistent methodology, conducting two consecutive BP measurements at a one-minute interval. The mean of the first and second measurements was considered the final BP value. BP control was defined by a systolic BP <140 mmHg and a diastolic BP <90 mmHg (12). Among patients who underwent re-examination, serum LDL-C concentrations and venous blood HbA1c levels were determined. LDL-C control was defined by an LDL-C level < 2.6 mmol/L (1,000 mg/L) (12). Glycemic control was defined by an HbA1c level ≤ 7% (12). For patients with a history of smoking and experiencing an ischemic stroke, smoking cessation was recommended. Similar recommendations were extended to alcohol consumption, suggesting abstinence or reduced intake. Lifestyle modifications such as moderate sodium reduction, increased potassium intake, and the use of potassium-containing salt were advocated for patients with ischemic stroke. Patients with diabetes were recommended to adhere to a diabetic diet. Patients with ischemic stroke were recommended to engage in at least three to four sessions of moderate-intensity exercise, such as brisk walking, lasting a minimum of 10 min per week. Alternatively, at least two sessions of aerobic exercise, such as brisk walking and jogging lasting at least 20 min, per week were recommended. These guidelines carry a Level I recommendation with Level B evidence (12).

### 2.4 Assess medication compliance

We defined compliance as the consistency in taking prescribed medications from the time of discharge up to 3, 6, and 12 months after stroke. The patients were also considered compliant if they switched to a different medication within the same therapeutic category (for example, altering antihypertensive medications within the same class). Non-compliance was attributed to situations where the prescribed discharge medication or alternative medications with similar effects were not taken or change your medication regimen on your own.

### 2.5 Statistical methods

Baseline characteristics are presented as medians with interquartile ranges (IQRs) for continuous variables and as frequencies and percentages for categorical variables. In the context of a mixed-model logistic regression, we used individual iterations for BP, LDL-C, and dichotomized HbA1c, using one at a time as dependent variables and time points as categorical variables, to calculate the proportion achieving treatment targets. In the assessment of the association between potential explanatory factors and the achievement of targets in patients adhering to prescribed medications, the model encompassed several covariates, namely age, sex, ethnicity, educational level, medical insurance coverage type, presence of coronary atherosclerotic heart disease, history of previous stroke, NIHSS score at discharge, infarct site (anterior, posterior, or multiple circulations), admission time, and treatment method (intravenous thrombolysis, vascular intervention, bridging therapy, and conventional treatment). Furthermore, a univariate analysis was performed for the type of medication used, smoking cessation, alcohol restriction, adoption of lifestyle modifications, and engagement in regular physical activities. We performed both unadjusted and adjusted analyses. The adjusted analysis considered adjusting for age, sex, ethnicity, educational level, and medical insurance coverage as demographic factors. Additionally, we adjusted for all variables with a significance level of *p* < 0.1 from both the demographic factor and univariate analyses. Odds ratios (ORs) and their corresponding 95% confidence intervals (CIs) are presented as our reporting metrics. Two-sided *p* values <0.05 were considered statistically significant. However, *p* values between 0.01 and 0.05 should be interpreted with caution due to multiple assumptions. Data analysis was performed using Stata SPSS version 26.

## 3 Results

### 3.1 Baseline data

A total of 1773 patients with acute ischemic stroke were initially included in the study. Patients who died during hospitalization (*n* = 63) and those discharged without medication (*n* = 138) were excluded, resulting in a final analysis cohort of 1,572 patients for analysis (Figure 1). Among these, 72% (*n* = 1,132) completed a three-month follow-up, with 84.27% (*n* = 954) participating in hospital re-examinations. Similarly, 67.11% (*n* = 1,055) attended the six-month follow-up, with 88.3% (*n* = 932) returning for hospital re-examination. For the 12-month follow-up, 58.33% (*n* = 917) were accounted for, and 87.56% (*n* = 803) of these patients came to the hospital for re-examination. The clinical characteristics of the study population are presented in Table 1. The median age of the participants was 65 years (IQR, 55–75), and 60.4% of them were male. At discharge, the median number of secondary prevention medications taken was 3 (IQR, 2–4), and all patients (100%) were prescribed at least one secondary prevention medication.

**Table 1:** Baseline characteristics of the study population (*n* of the 1752 patients eligible for analysis).

Variables	Overall (*n* = 1752)
Age, median (IQR), years	65 (55.75)
Male, (*n*%)	950 (60.4%)
Ethinicity (Han), (*n*%)	1,482 (94.3%)
Health insurance, (*n*%)	
NRCMS	921 (58.6%)
UBMIS	391 (24.9%)
Self-payment	260 (16.5%)
Smoking	647 (41.2%)
Drinking	338 (21.5%)
Residence (Rural), (*n*%)	1,079 (68.6%)
Education level, *n* (%)	
Middle school or above	671 (42.7%)
Elementary or below	901 (57.3%)
Hypertension	1,080 (68.7%)
Diabetes	367 (23.3%)
Dislipidaemia	655 (41.7%)
Atrial fibrillation	37 (2.4%)
Carotid plaque	1,162 (73.9%)
Coronary artery disease	241 (15.3%)
Previous stroke	205 (13.0%)
Days of hospitalization, median (IQR)	10(7,14)
Time of onset, (*n*%)	
≤6 h	300 (19.1%)
>6 h,≤24 h	474 (30.2%)
>24 h,≤72 h	502 (31.9%)
>72 h,≤7 days	296 (18.8%)
Treatment options, (*n*%)	
Intravenous thrombolysis	163 (10.4%)
Endovascular treatment	9 (0.6%)
Bridging therapy	13 (0.8%)
Conventional treatment	1,387 (88.2%)
Infarction site, (*n*%)	
Anterior circulation infarction	1,232 (78.4%)
Posterior circulation infarction	314 (20.0%)
Infarcts in multiple cerebral circulations	26 (1.7%)
Admission NIHSS score, median (IQR)	3(2,5)
NIHSS score at discharge, median (IQR)	2(1,4)
Type of medication, median (IQR)	3(2,4)

NRCMS, new rural cooperative medical schemes; UBMIS, urban basic medical insurance schemes; NIHSS, National Institutes of Health Stroke Scale.

### 3.2 BP control

Out of the 1,572 patients with acute ischemic stroke, 68.7% (*n* = 1,080) were diagnosed with hypertension. All patients were prescribed antihypertensive medication upon discharge. For the follow-up assessments, 60.0% (*n* = 954), 59.2% (*n* = 932), and 51% (*n* = 803) of the patients had their BP measured at the outpatient clinic at 3, 6, and 12-month intervals, respectively. Notably, 50.7, 51.8, and 50.6% achieved the BP control targets at 3, 6, and 12 months, respectively, across the entire patient population. The corresponding rates of achieving BP control among patients taking antihypertensive medications were 49.1, 53.2, and 53.6% (Table 2).

**Table 2:** Proportions achieving vascular risk factor control at 3 months, at 6 months and 12 months.

	All patients			Patients prescribed pharmacotherapy		
	*n* Probability (%) 95% CI (%)			*n* Probability (%) 95% CI (%)		
3 months						
Blood pressure control	484	50.70%	0.48–0.54	249	49.11%	0.02–0.11
LDL cholesterol control	491	51.50%	0.48–0.55	378	71.86%	0.03–0.08
Glycemic control	141	48.10%	0.42–0.54	135	60.81%	0.02–0.15
6 months						
Blood pressure control	483	51.80%	0.49–0.55	272	53.23%	0.02–0.11
LDL cholesterol control	554	59.40%	0.56–0.63	414	71.01%	0.12–0.25
Glycemic control	125	44.00%	0.38–0.50	119	62.00%	0.01–0.10
12 months						
Blood pressure control	406	50.60%	0.47–0.54	216	53.60%	0.03–0.12
LDL cholesterol control	406	50.60%	0.47–0.54	326	68.92%	0.03–0.08
Glycemic control	89	48.40%	0.33–0.46	84	57.53%	0.00–0.04

Based on mixed model logistic regression with time point as categorical covariate and patient as random effect. Blood pressure control, Blood pressure (BP) < 140/90 mmHg; LDL cholesterol control, LDL cholesterol < 2.6 mmol/L; Glycemic control, HbA1c ≤ 7%.

### 3.3 LDL control

All the 1752 patients enrolled, 655 had lipid metabolism disorders (Table 1), and all of these patients were taking statins in this study. LDL-C was measured in 60.0, 59.2, and 51% of patients during the 3, 6, and 12-month follow-up periods, respectively. Throughout these timeframes, the criteria for achieving LDL-C control were satisfied by 51.5, 59.4, and 50.6% of all patients. Notably, patients under lipid-lowering medications achieved target LDL-C control rates of 71.9, 66.75, and 68.9% for the respective time points (Table 2).

### 3.4 Glycemic control

Among the patients with acute ischemic strokes, 23.3% (*n* = 367) were diagnosed with diabetes mellitus and received glucose control medication upon discharge. Among these patients with diabetes mellitus, HbA1c measurements were obtained for 79.8, 86.6, and 58.5% at 3, 6, and 12 months, respectively. Hemoglobin control targets were achieved in 48.1, 44.0, and 48.4% of patients at 3, 6, and 12 months, respectively. The corresponding values for participants on antidiabetic medications were 60.8, 62, and 57.5% (Table 2).

### 3.5 Optimal control of all targets

A total of 60.7% (*n* = 954), 59.2% (*n* = 932), and 51% (*n* = 803) of patients completed three sets of BP, LDL-C, and HbA1c measurements at 3, 6, and 12 months. Of these, 29.6% (*n* = 282), 29.0% (*n* = 271), and 27.1% (*n* = 218) patients, respectively, exhibited optimal control across all three risk factor targets. Among patients who smoked, 38.9% (250 out of 643), 30.5% (177 out of 581), and 28.2% (154 out of 546) continued smoking at 3, 6, and 12 months, respectively. For those who consumed alcohol before the stroke, 40.1% (135 out of 337), 30.7% (88 out of 287), and 25.5% (69 out of 271) remained unrestricted at the same intervals. Around 51.5% (810 out of 1,572), 56.6% (824 out of 1,457), and 62.1% (881 out of 1,418) maintained an unchanged lifestyle at 3, 6, and 12 months, respectively. In patients without limb movement disorders, irregular physical activity was observed in 60.5% (903 out of 1,455), 65.5% (881 out of 1,345), and 68.9% (900 out of 1,306) at 3, 6, and 12 months, respectively.

### 3.6 Factors associated with vascular risk factor control

Age, sex, ethnicity, educational level, medical insurance coverage type, presence of coronary atherosclerotic heart disease, history of previous stroke, NIHSS score, infarct site (anterior, posterior, or multiple circulations), admission time, and treatment methods (intravenous thrombolysis, vascular intervention, bridging therapy, and conventional treatment) were subjected to univariate analysis (Table 3). This analysis was performed for variables such as medication type, smoking cessation, alcohol restriction, lifestyle modifications, and engagement in regular physical activity. Among the factors influencing BP control with antihypertensive medications, age demonstrated a *p* value of <0.001, while ethnicity, education level, coronary atherosclerotic heart disease, smoking cessation, alcohol restriction, and lifestyle modifications all showed *p* values of <0.05. In terms of factors impacting blood glucose control, *p* values were < 0.001 for both lifestyle modification and regular physical activity (Table 3).

**Table 3:** Univariate analysis of vascular risk factor control in patients taking medication in 12-month.

	Blood pressure control 95% CI (%) *p* value	Glycemic control 95% CI (%) *p* value	LDL cholesterol control 95% CI (%) *p* value
Sex (female)	0.80 (0.54, 1.19) 0.2697	0.74 (0.43, 1.30) 0.2943	0.73 (0.49, 1.09) 0.1271
Age	0.96 (0.95, 0.98) < 0.0001	0.99 (0.96, 1.01) 0.1789	0.98 (0.96, 1.00) 0.0137
Days of hospitalization	0.99 (0.96, 1.02) 0.5216	0.98 (0.93, 1.02) 0.2931	1.03 (1.00, 1.07) 0.0675
Ethinicity (National minority)	4.16 (1.38, 12.52) 0.0112	1.32 (0.39,1.0 4.47) 0.6568	0.90 (0.33, 2.44) 0.8331
Health insurance			
NRCMS	Reference	Reference	Reference
UBMIS	0.90 (0.56, 1.46) 0.6732	0.50 (0.24, 1.01) 0.0519	1.05 (0.64, 1.74) 0.8462
Self-payment	0.69 (0.40, 1.17) 0.1661	0.73 (0.33, 1.60) 0.4320	0.68 (0.40, 1.15) 0.1456
Residence (Rural)	1.26 (0.84, 1.89) 0.2702	0.79 (0.44, 1.41) 0.4294	1.00 (0.66, 1.52) 0.9878
Education level (elementary or below)	0.62 (0.42, 0.93) 0.0195	0.74 (0.42, 1.28) 0.2749	0.86 (0.58, 1.28) 0.4571
Coronary artery disease	1.80 (1.02, 3.17) 0.0436	1.17 (0.51, 2.68) 0.7183	1.68 (0.96, 2.92) 0.0667
Previous stroke	1.00 (0.52, 1.92) 0.9935	0.60 (0.32, 1.13) 0.1154	0.63 (0.41, 0.95) 0.0276
Time of onset, (*n*%)			
≤6 h	Reference	Reference	Reference
>6 h,≤24 h	1.93 (1.06, 3.54) 0.0329	1.05 (0.43, 2.60) 0.9122	0.80 (0.45, 1.43) 0.4495
>24 h,≤72 h	1.38 (0.77, 2.48) 0.2749	1.61 (0.67, 3.89) 0.2909	0.92 (0.52, 1.63) 0.7664
>72 h,≤7 days	2.06 (1.06, 4.01) 0.0330	2.20 (0.85, 5.71) 0.1049	1.14 (0.58, 2.23) 0.7107
Treatment options, (*n*%)			
Intravenous thrombolysis	Reference	Reference	Reference
Endovascular treatment	2.32 (0.19, 27.59) 0.5065	1.63 (0.09, 29.78) 0.7435	1439453.22 (0.00, Inf) 0.9826
Bridging therapy	0.00 (0.00, Inf) 0.9800	0.00 (0.00, Inf) 0.9873	0.38 (0.06, 2.53) 0.3135
Conventional treatmen	1.39 (0.72, 2.65) 0.3244	1.05 (0.42, 2.66) 0.9168	0.51 (0.26, 1.02) 0.0575
Infarction site, (*n*%)			
Anterior circulation infarction	Reference	Reference	Reference
Posterior cicirculation infarction	0.83 (0.52, 1.34) 0.4484	1.27 (0.66, 2.46) 0.4791	1.13 (0.69, 1.86) 0.6257
Infarcts in multiple cerebal circulations	0.83 (0.05, 13.41) 0.8964	1.65 (0.10, 26.86) 0.7247	1.39 (0.14, 13.49) 0.7769
NIHSS Scoreat discharge	0.97 (0.86, 1.09) 0.5914	0.87 (0.74, 1.02) 0.0807	0.91 (0.84, 0.99) 0.0336
Smoking			
No smoking	Reference	Reference	Reference
Quit smoking	2.52 (1.58, 4.01) < 0.0001	1.28 (0.69, 2.36) 0.4293	1.25 (0.84, 1.87) 0.2740
Still smoking	0.70 (0.32, 1.56) 0.3887	0.26 (0.06, 1.21) 0.0859	1.74 (0.35, 8.55) 0.4950
Drinking			
No drinking	Reference	Reference	Reference
Quit drinking	2.31 (1.24, 4.29) 0.0081	1.18 (0.51, 2.71) 0.7005	1.89 (1.05, 3.40) 0.0343
Still drinking	0.59 (0.19, 1.85) 0.3669	1.61 (0.22, 11.66) 0.6396	0.19 (0.07, 0.56) 0.0025
No lifestyle changes	0.19 (0.12, 0.30) < 0.0001	0.06 (0.03, 0.13) < 0.0001	0.32 (0.21, 0.48) < 0.0001
Lack of regular physical activity	0.86 (0.57, 1.29) 0.4549	0.31 (0.17, 0.55) < 0.0001	0.48 (0.31, 0.74) 0.0009

Based on mixed model logistic regression with time point as categorical covariate and patient as random effect. NIHSS, National Institutes of Health Stroke Scale; NRCMS, new rural cooperative medical schemes; UBMIS, urban basic medical insurance schemes; Blood pressure control, Blood pressure (BP) < 140/90 mmHg; LDL cholesterol control, LDL cholesterol < 2.6 mmol/L; Glycemic control,HbA1c ≤ 7%.

Among the factors influencing lipid control with lipid-lowering medications, the *p* values for both alcohol restriction and regular physical activity were < 0.001. Additionally, the *p* values for age, lifestyle modification, previous history of stroke, and NIHSS score at discharge were < 0.05. Thus, we included all these factors in the logistic regression. In Model 1, we adjusted for demographic factors including sex, age, ethnicity, education, address, and medical insurance coverage type. Model 2, on the other hand, adjusted all variables (Table 4) with a *p*-value of <0.1 from the univariate analysis. This included factors such as coronary atherosclerotic heart disease, previous history of stroke, NIHSS score at the hospital, smoking cessation, alcohol restriction, lifestyle modifications, and regular physical activity, in conjunction with demographic factors.

**Table 4:** Mixed model logistic regression with vascular risk factor control as dependent variable, for participants prescribed pharmacotherapy by 12 months.

		Model 1		Model 2	
		Adjusted HR/OR (95% CI)	*p* value	Adjusted HR/OR (95% CI)	*p* value
Age	Blood pressure control	0.96 (0.94, 0.98)	<0.01	0.96 (0.93, 0.98)	<0.01
	Glycemic control	0.99 (0.97, 1.02)	0.65	1.02 (0.98, 1.06)	0.34
	LDL cholesterol control	0.98 (0.96, 1.00)	0.03	0.99 (0.97, 1.01)	0.44
Ethinicity (National minority)	Blood pressure control	3.93 (1.25, 12.38)	0.02	4.23 (1.19, 15.09)	0.03
	Glycemic control	1.36 (0.37, 4.93)	0.64	0.92 (0.10, 8.45)	0.94
	LDL cholesterol control	0.84 (0.30, 2.35)	0.74	0.71 (0.22, 2.26)	0.56
Coronary artery disease	Blood pressure control	0.5 (0.2, 0.8)	0.01	0.5 (0.3, 1.0)	0.05
	Glycemic control	0.8 (0.3, 1.9)	0.59	0.6 (0.2, 2.2)	0.47
	LDL cholesterol control	0.5 (0.3, 1.0)	0.04	0.6 (0.3, 1.2)	0.14
Previous stroke	Blood pressure control	0.8 (0.4, 1.7)	0.61	1.5 (0.7, 3.3)	0.31
	Glycemic control	1.9 (0.9, 3.9)	0.08	6.3 (2.3, 16.8)	<0.01
	LDL cholesterol control	1.7 (1.1, 2.7)	0.02	1.7 (1.0, 2.8)	0.03
NIHSS score at discharge, median	Blood pressure control	1.0 (0.9, 1.2)	0.63	1.0 (0.9, 1.2)	0.91
	Glycemic control	1.2 (1.0, 1.4)	0.06	1.2 (1.0, 1.5)	0.06
	LDL cholesterol control	1.1 (1.0, 1.2)	0.03	1.1 (1.0, 1.2)	0.08
tobacco cessation	Blood pressure control	0.5 (0.3, 0.8)	0.01	0.5 (0.3, 0.8)	0.01
	Glycemic control	1.0 (0.5, 2.2)	0.99	1.1 (0.3, 3.3)	0.92
	LDL cholesterol control	1.1 (0.6, 1.9)	0.74	1.1 (0.6, 2.0)	0.71
Still drinking	Blood pressure control	1.9 (0.6, 6.5)	0.31	0.8 (0.2, 3.1)	0.76
	Glycemic control	0.7 (0.1, 5.6)	0.77	0.1 (0.0, 1.1)	0.06
	LDL cholesterol control	6.9 (2.3, 21.0)	<0.01	3.3 (1.0, 11.1)	0.05
No lifestyle changes	Blood pressure control	0.19 (0.12, 0.30)	<0.01	0.19 (0.11, 0.31)	<0.01
	Glycemic control	0.06 (0.03, 0.13)	<0.01	0.03 (0.01, 0.08)	<0.01
	LDL cholesterol control	0.33 (0.21, 0.50)	<0.01	0.26 (0.16, 0.42)	<0.01
Lack of regular physical activity	Blood pressure control	1.25 (0.79, 1.97)	0.33	1.25 (0.79, 1.97)	0.06
	Glycemic control	0.29 (0.16, 0.54)	<0.01	0.14 (0.06, 0.36)	<0.01
	LDL cholesterol control	0.53 (0.34, 0.85)	0.01	0.55 (0.33, 0.90)	0.02

Model 1, Adjustment for sex, age, ethnicity, education, address, type of health insurance demographic factors.

Model 2 In addition to adjusting for demographic factors, all variables with *p* < 0.1 in the univariate analysis were adjusted, including coronary atherosclerotic heart disease, history of previous stroke, discharge NIHSS score, smoking cessation, alcohol cessation, lifestyle changes, and regular physical activity.

NIHSS, National Institutes of Health Stroke Scale; Blood pressure control, Blood pressure (BP) < 140/90 mmHg; LDL cholesterol control, LDL cholesterol < 2.6 mmol/L; Glycemic control, HbA1c ≤ 7%.

Upon analysis, the results showed that after adjusting for sex, ethnicity, education, address, and medical insurance coverage type, a statistically significant correlation between age and BP control remained (OR, 0.96; 95% CI, 0.94–0.98; *p* < 0.0001). After further adjustment for the demographic factors, a statistically significant correlation was also observed between coronary atherosclerotic heart disease, previous history of stroke, NIHSS score in the hospital, smoking cessation, alcohol restriction, lifestyle modifications, and regular physical activity (OR, 0.96; 95% CI, 0.94–0.98; *p* < 0.0001). In Model 1, ethnic minorities had a lower BP control ratio compared with the Han population (OR, 3.93; 95% CI, 1.25–12.38; *p* = 0.02). After further adjustment for factors like coronary atherosclerotic heart disease, previous history of stroke, NIHSS score at the hospital, smoking cessation, alcohol restriction, lifestyle modifications, and regular physical activity, the association between ethnic group and achievement of BP control remained statistically significant (OR, 4.23; 95% CI, 1.19–15.09; *p* = 0.02).

The correlation between having coronary atherosclerotic heart disease and achieving BP control showed a reduction. In Model 1, the OR was 0.5 (95% CI, 0.2–0.8, *p* = 0.01), while in Model 2, it was 0.5 (95% CI, 0.3–1.0, *p* = 0.05). Similarly, the history of a previous stroke and lipid control exhibited statistical significance. In Model 1, the OR was 1.7 (95% CI, 1.1–2.7, *p* = 0.01), and in Model 2, it was 1.7 (95% CI, 1.0–2.8, *p* = 0.03). Furthermore, when adjusting for age, sex, ethnicity, education, address, and medical insurance type, non-drinking versus unrestricted drinking was statistically significant with respect to lipid control (OR, 6.9; 95% CI, 2.3–21; *p* < 0.01). After adjusting for demographic factors and coronary atherosclerotic heart disease, previous history of stroke, NIHSS score at the hospital, smoking cessation, lifestyle modifications, and regular physical activity, unrestricted alcohol consumption maintained a statistically significant association with lipid control (OR, 3.3; 95% CI, 1.0–11.1; *p* = 0.05).

After adjusting for sex, ethnicity, education, address, medical insurance coverage type, lifestyle modifications, and BP control, the OR was 0.19 (95% CI, 0.12–0.30, *p* < 0.01). Likewise, for glycemic control, the OR was 0.06 (95% CI, 0.03–0.13, *p* < 0.01), and for lipid control, it was 0.33 (95% CI, 0.21–0.50, *p* < 0.01), all adjusted for demographic factors. Further adjustments, while considering coronary atherosclerotic heart disease, previous history of stroke, NIHSS score in hospital, smoking cessation, alcohol restriction, lifestyle modifications, and regular physical activity, still exhibited significant correlations between lifestyle modifications and BP control (OR, 0.19; 95% CI, 0.12–0.30; *p* < 0.01), glycemic control (OR, 0.03; 95% CI, 0.01–0.08; *p* < 0.01) and lipid control (OR, 0.26; 95% CI, 0.16–0.42; *p* < 0.01). Compared with regular physical activity, irregular physical activity demonstrated a lower odds ratio for glycemic and lipid controls. Adjusted for demographic factors, the OR for glycemic control was 0.29 (95% CI, 0.16–0.54, *p* < 0.01), and for lipid control, it was 0.53 (95% CI, 0.34–0.85, *p* < 0.01). While adjusting for demographic factors and coronary atherosclerotic heart disease, previous history of stroke, NIHSS score, smoking cessation, alcohol restriction, and lifestyle modifications, the OR for glycemic control was 0.14 (95% CI, 0.06–0.36, *p* < 0.01), and for lipid control, it was 0.55 (95% CI, 0.33–0.90, *p* = 0.01).

Details regarding secondary prevention medication compliance are shown in Table 5.

**Table 5:** Persistence to secondary preventive medication at 3 months, 6 months and 12 months for participants.

	Medication prescribed at discharge *n* (%)	Persistent at 3 months *n* (%)	Persistent at 6 months *n* (%)	Persistent at 12 months *n* (%)
Antihypertensive drugs	1,080(68.7)	798(73.9)	703(69.3)	622(65.3)
Antidiabetic drugs	367(23.3)	277(75.7)	244(70.1)	230(68.9)
Anticoagulation drugs	37(2.4)	26(70.3)	20(62.5)	17(56.7)
Antithrombotic drugs	1,535(97.6)	1,097(71.5)	971(68.1)	862(63.4)
Lipid lowering drugs	1,257(80)	896(66.6)	811(64.4)	710(59.2)

### 3.7 Persistence to medication prescribed at discharge

In this study, the patients took prescribed medications from the time of discharge up to 3, 6, and 12 months after stroke and the patients were also considered compliant if they switched to a different medication within the same therapeutic category (for example, altering antihypertensive medications within the same class). Non-compliance was attributed to situations where the prescribed discharge medication or alternative medications with similar effects were not taken or change your medication regimen on your own.

Around 68.7% (*n* = 1,080) of patients were discharged while being prescribed antihypertensive medications, while 80% (*n* = 1,257) were prescribed lipid-lowering medications. Moreover, 97.6% (*n* = 1,535) received antiplatelet aggregation medications, 23.3% (*n* = 367) were on antidiabetic medications, and 2.4% (*n* = 37) were prescribed anticoagulants. At 3, 6, and 12 months, 73.9% (*n* = 798), 69.3% (*n* = 703), and 65.3% (*n* = 622) of patients were taking antihypertensive medications; 66.6% (*n* = 896), 64.4% (*n* = 811), and 59.2% (*n* = 710) of patients were on lipid-lowering medications; and 75.7% (*n* = 277), 70.1% (*n* = 244), and 68.9% (*n* = 230) of patients were on hypoglycemic medications, respectively. Regarding patients taking antiplatelet aggregation medications, 71.5% (*n* = 1,097), 68.1% (*n* = 971), and 63.4% (*n* = 862) adhered to their regimen at 3, 6, and 12 months, respectively. For patients on hypolipidemic medications, the percentages were 66.6% (*n* = 896), 64.4% (*n* = 811), and 59.2% (*n* = 710) at the same intervals. Finally, patients prescribed anticoagulant medications demonstrated compliance rates of 70.3% (*n* = 26), 62.5% (*n* = 20), and 56.7% (*n* = 17) at 3, 6, and 12 months, respectively.

Medication compliance declined progressively over time. The results demonstrated the utilization of antiplatelet, lipid-lowering, antihypertensive, hypoglycemic, and anticoagulant medications among patients with atrial fibrillation. At 3 months after discharge, the compliance of these medications, ranked from highest to lowest, was hypoglycemic, antihypertensive, antiplatelet aggregation, anticoagulant, and lipid-lowering medications. Similarly, 6 months after discharge, the order of medication compliance from highest to lowest remained consistent, with hypoglycemic, antihypertensive, antiplatelet aggregation, lipid-lowering, and anticoagulant medications. The ranking of these secondary prevention medications remained unchanged at 12 months, mirroring the trend observed at 6 months.

## 4 Discussion

Standard secondary protocols for vascular risk factor control have demonstrated the potential to significantly reduce the risk (26). Remarkably, around 80% of ischemic strokes could be prevented through effective control of BP, blood sugar, blood lipids, and antiplatelet aggregation, and anticoagulant therapies (27). However, the current utilization levels of vascular risk factor control fall short of ideal. Most evidence-based or guideline-endorsed stroke prevention tools are underutilized (28). Many previous studies (24, 25, 29, 30) have focused on compliance with secondary prevention medications and the influencing factors associated with their usage. However, only a limited number of studies have investigated factors associated with vascular risk factor control. Our findings highlight a concerning reality: not only is compliance with secondary prevention medications lacking, but even among patients with ischemic stroke who regularly take these medications, effective vascular risk factor control remains suboptimal. A large proportion of these patients fail to attain the guideline-recommended targets of BP, LDL-C, and HbA1c. At 3, 6, and 12-month follow-up. Accordingly, a mere 29.6, 29.0, and 27.1% demonstrated optimal control across all three risk factors.

Upon 12-month follow-up, we found that age, ethnicity, history of coronary atherosclerotic heart disease, smoking cessation, and lifestyle modifications were associated with the achievement of BP control. Moreover, a previous history of stroke, NIHSS scores at discharge, alcohol consumption, lifestyle modifications, and regular physical activity were associated with lipid control. Additionally, previous history of stroke, lifestyle modifications, and regular physical activity were associated with the achievement of glycemic control. For compliance with secondary prevention medications, a clear trend of decreasing overall compliance was observed as the follow-up period extended. This trend was especially pronounced for patients who did not attend the hospital for re-examination or telephone follow-ups, highlighting poorer medication compliance among this subgroup.

According to the discharge medication type, the order of prevalence from highest to lowest was antiplatelet, lipid-lowering, antihypertensive, hypoglycemic, and anticoagulant medications, especially among patients with atrial fibrillation. Three months after discharge, the compliance of these medications, from highest to lowest, was hypoglycemic, antihypertensive, antiplatelet aggregation, anticoagulant, and lipid-lowering medications. Six months after discharge, the compliance of the above medications, from highest to lowest, was hypoglycemic, antihypertensive, antiplatelet aggregation, lipid-lowering, and anticoagulant medications. This pattern persisted at the 12-month follow-up, mirroring the same trend at the six-month interval.

Currently, in China, healthcare professionals place significant emphasis on acute management and secondary prevention measures as recommended by the guidelines for acute ischemic stroke (29, 30). However, there remains dissatisfaction with the vascular risk factor control treatment for patients with stroke following their discharge. In recent years, Western countries have experienced a substantial reduction in the recurrence rates of stroke (31–33), while in China, the recurrence rates of stroke remain higher than the average seen in Western countries (34). A key contributor to this elevated recurrence rate of stroke is poor compliance with secondary stroke prevention guidelines and inadequate risk factor control (35, 36). The persistence of uncontrolled vascular risk factors places many patients at continued risk of recurrent stroke. To effectively lower the recurrence rate of stroke, it is imperative to increase compliance with vascular risk factor control, improve compliance with guidelines, and bridge the gap between clinical practice and evidence-based medicine to reduce the recurrence rate of stroke and preserve lives.

Our survey has underscored the limited attainment rates of vascular risk factor control as prescribed by evidence-based medical guidelines for secondary prevention. The factors contributing to this suboptimal level of achievement are likely multifaceted. Age, ethnicity, and a history of coronary atherosclerotic heart disease are closely related to the BP control standard through analysis of factors affecting patients’ vascular risk factor control standard (37). This study, in particular, sheds light on the positive correlation between age and hypertension. Notably, the rate of achieving the standard BP control rate tends to be lower in elderly patients (11). Yang et al. (38) showed that ethnic groups were associated with the achievement of hypertension control, and our study showed that ethnic minorities had a higher rate of BP control than Han nationalities, which may be related to better compliance among ethnic minorities.

In summary, it is crucial to give increased attention to the treatment and monitoring of hypertension in elderly patients, individuals of Han nationality, and those with a history of coronary atherosclerotic heart disease to improve the compliance rate. Additionally, it has been found that not imposing restrictions on alcohol consumption correlates with achieving effective lipid control, and alcohol consumption promotes achieving LDL targets, consistent with relevant studies (39, 40). Although alcohol consumption promotes lowering LDL levels, its potential impact on the recurrence of stroke cannot be neglected. Both the guidelines of the European Society of Hypertension and the European Society of Cardiology and relevant studies emphasize the positive influence of a healthy lifestyle on hypertension management (41, 42). Moreover, lifestyle modifications prove beneficial for diabetes control (43, 44). Regular physical activity is highlighted as a means to reduce insulin resistance and enhance glycemic control. This stance is reinforced by the 2020 version of the Chinese Guidelines for the Prevention and Treatment of Type 2 Diabetes (45), which specifies exercise therapy as a fundamental approach to controlling hyperglycemia. As per the Chinese lipid management guideline (46–48), lipid-lowering therapy prioritizes a healthy lifestyle, including a balanced diet and a gradual increase in physical activity. Our survey findings indicated a consistent correlation between lifestyle modifications among patients with ischemic stroke and the management of various factors, such as BP, blood glucose, and blood lipid levels. Notably, engaging in regular physical activity demonstrated a positive impact on both blood glucose and blood lipid levels. Therefore, patients should be encouraged to adopt a healthier lifestyle and encourage autonomous mobility, particularly by promoting regular physical activity. These efforts are pivotal in effectively controlling vascular risk factors, thereby striving to achieve the target of reducing the recurrence rate of stroke.

Compliance with secondary prevention medications has gradually declined over time, especially with antiplatelet, lipid-lowering, and anticoagulant medications. Compared with antihypertensive and hypoglycemic medications, patients might possess a lesser awareness of this class of medications. Hence, alongside improving patients’ awareness of the importance of vascular risk factor control, it is necessary to promote the importance of utilizing antiplatelet aggregation, lipid-lowering, and anticoagulant medications among patients with atrial fibrillation to improve compliance with secondary prevention medications and reduce the recurrence rate of stroke.

This study has some limitations that warrant consideration. Firstly, it is possible that the individuals who participate in outpatient follow-up might consist of patients with worse vascular risk factor control, potentially leading to an overestimation of the compliance rate regarding vascular risk factor control. Additionally, the assessment of compliance relied on telephone interviews for certain aspects of the study. While there was a high level of concurrence between compliance reported during telephone follow-ups and the analysis of medication data, these findings might have been compromised by the subjective responses of patients, introducing the potential for recall and information biases.

## 5 Conclusion

Our results showed that, among patients undergoing medication, the achievement rate of vascular risk factor control was unsatisfactory. This emphasizes a critical aspect: in the context of secondary prevention of stroke, attention should not solely be focused on the compliance of secondary prevention medications but equally, if not more, on vascular risk factor control. This becomes the key to prevention, especially in the case of an increasing stroke burden.

## 6 Limitations

Although the article included a large sample, the overall age of the patients was young as well as the stroke was mild, the study included data related to the period from January 2019 to December 2022, which is a short duration. With a follow-up rate of just 50 percent within 1 year, this study has a high rate of loss to follow-up, which may have affected the results. However, this study is still under continuous observation and follow-up, and in the future, we will also strengthen the health education of patients and raise their awareness of disease treatment and prevention to reduce the loss of follow-up rate.

## Data availability statement

The original contributions presented in the study are included in the article/supplementary material, further inquiries can be directed to the corresponding author.

## Ethics statement

The studies involving humans were approved by the Ethics Committee of Zhejiang Provincial People's Hospital Bijie Hospital, Bijie, China (Approval number: 3-1; Year: 2023). The studies were conducted in accordance with the local legislation and institutional requirements. Written informed consent for participation was not required from the participants or the participants' legal guardians/next of kin in accordance with the national legislation and institutional requirements.

## Author contributions

YC: Writing – review & editing, Writing – original draft. YZ: Writing – review & editing, Methodology. LJ: Writing – original draft, Methodology, Data curation, Writing – review & editing. YL: Writing – original draft, Investigation, Conceptualization. XD: Writing – review & editing, Data curation, Conceptualization. WJ: Writing – original draft, Supervision, Formal analysis. CX: Writing – review & editing, Supervision, Formal analysis. DH: Writing – original draft, Resources, Project administration.
